# The diagnosis of diabetic striatopathy should be made only after careful exclusion of all possible differential diagnoses

**DOI:** 10.51866/lte.26-00010

**Published:** 2026-08-27

**Authors:** Sounira Mehri, Josef Finsterer

**Affiliations:** 1 Biochemistry Laboratory, LR12ES05 "Nutrition-Functional Foods and Vascular Health", Faculty of Medicine, Monastir, Tunisia.; 2 Neurology Dpt., Neurology & Neurophysiology Center, Vienna, Austria.

**Keywords:** Diabetes mellitus, Corpus striatum, Magnetic resonance imaging, Chorea

**Dear Editor,**

We read with interest the article by Rahim et al. about an 87-year-old female patient with longstanding and poorly controlled diabetes mellitus. She was diagnosed with diabetic striatopathy (DS) after developing choreatiform movements in her right arm, twitching in her right leg and twitching of the right corner of her mouth 3 weeks prior to admission.^1^ The symptoms resolved completely within 3 weeks of improved diabetes control.^1^ The study is interesting, but some points warrant discussion.

First, other differential diagnoses of DS were not adequately ruled out. The most important differential diagnoses for DS include cerebrovascular events such as basal ganglia infarction or haemorrhage, osmotic demyelination resulting from overly rapid correction of hyperglycaemia, infectious diseases (e.g. Japanese encephalitis), tumours (e.g. glioma or lymphoma), immunological diseases (e.g. lupus erythematosus), neurodegenerative diseases (e.g. Parkinson’s disease, Huntington’s disease or Wilson’s disease), Fahr’s disease and mitochondrial diseases.^2^ These differential diagnoses must be carefully excluded before the clinical presentation is attributed to DS.

Second, the differential diagnoses of chorea were also not adequately ruled out.^1^ The genetic causes of chorea that should have been excluded include Huntington’s disease; Huntington-like disorders 1–3; hereditary prion diseases; spinocerebellar ataxia types 1, 3 and 17; neuroacanthocytosis; dentatorubral–pallidoluysian atrophy; cerebral iron deposition disorders; Wilson’s disease; benign hereditary chorea; Friedreich’s ataxia; and MID.^3^ The acquired causes that should have been excluded include vascular diseases, post-infectious autoimmune diseases of the central nervous system, medications, systemic lupus erythematosus, antiphospholipid syndrome, thyrotoxicosis, AIDS, chorea gravidarum and polycythaemia vera.^3^

Third, the patient did not undergo magnetic resonance imaging (MRI).^1^ MRI would have been essential to assess whether the described hyperdensity was due to cytotoxic oedema, vasogenic oedema, an epiphenomenon of a seizure, adult-onset Leigh syndrome, calcifications or haemorrhage. It would also have been helpful in determining whether the contralateral side was affected, as the computed tomography (CT) scan in Figure 1 suggests that the right side was also mildly involved. Typically, DS presents on MRI with T1-weighted hyperintensity, diffusion-wieghted imaging hyperintensity and T2-weighted hypointensity.^4^ Magnetic resonance spectroscopy would also have been useful to rule out or confirm the differential diagnoses. It could be helpful in excluding glioma (high N-acetyl aspartate peak and low choline peak) and adult-onset Leigh syndrome (lactate peak).

Fourth, no electroencephalography was recorded upon admission.^1^ Since the patient also exhibited twitching in the area of the right mouth and ankle joint, and hyperglycaemia can be associated with focal or generalised seizures or focal or generalised status epilepticus,^5^ it is crucial that epileptic activity is ruled out as the cause of clinical symptoms.

Fifth, no cerebrospinal fluid (CSF) analysis was performed on the patient.^1^ Testing the CSF for infectious agents, particularly neurotropic viruses, and for antibodies against autoimmune encephalitis would have been essential to rule out whether the radiological findings were due to focal encephalitis.

Sixth, the temporal course was inadequately documented.^1^ It would have been helpful to document the exact start date of hyperglycaemia treatment and the time of the CT scan to assess whether the imaging findings were due to a rapid normalisation of hyperglycaemia. If treatment was initiated before the CT scan, it cannot be ruled out that the hyperdensity in the left striatum was simply due to a premature normalisation of blood glucose levels.

Seventh, there is a discrepancy between the statement that muscle tone was normal during the clinical examination and the statement that dystonic movements were present.^1^ Dystonia is usually associated with increased muscle tone.^6^ Furthermore, it is unclear why no coordination disorder was described in the status report, even though the patient was simultaneously showing choreatic movements.

Eighth, the patient’s regular home medications were not listed.^1^ Since medications can trigger chorea,^7^ it is crucial to know which medications the patient was taking regularly. The medications that can cause chorea include dopamine receptor blockers, dopaminergic drugs, amphetamines, methylphenidate, valproic acid, carbamazepine, phenytoin and lamotrigine.

Finally, it is difficult to assess which of the values listed in Table 1 are normal and which are pathological, as no reference values were provided. It should also be explained what was meant by ‘osmotic symptoms’ and whether the clinical improvement was due to the use of risperidone or to antidiabetic therapy.
